# Pseudoaneurysm with a fistula to the right ventricle late after surgical repair of type A aortic dissection in a patient with systemic lupus erythematosus

**DOI:** 10.1186/s13019-022-01827-5

**Published:** 2022-04-27

**Authors:** Akie Shimada, Taira Yamamoto, Daisuke Endo, Kousuke Nishida, Satoshi Matsushita, Tohru Asai, Atsushi Amano

**Affiliations:** 1 Department of Cardiovascular Surgery, Juntendo University, Bunkyo-Ku, Tokyo, Japan; 2 Department of Cardiovascular Surgery, Nerima Hospital, Juntendo University, 3-1-10, Takanodai, Nerima-ku, Tokyo, 177-8521 Japan

**Keywords:** Aortic dissection, Systemic lupus erythematosus, Sjogren’s syndrome, Pseudoaneurysm, Fistula, Echocardiography-gated three-dimensional computed tomography

## Abstract

**Background:**

Pseudoaneurysm with a shunt to the right ventricle after aortic repair for acute aortic dissection is an extremely rare and life-threatening condition. Surgical treatment is unavoidable, but surgery is complicated, and there are some pitfalls. This study describes the reoperation performed in a patient at a high surgical risk by clarifying the shunt site using multimodality imaging before surgery.

**Case presentation:**

A 69-year-old woman with a history of systemic lupus erythematosus (SLE) and Sjogren’s syndrome presented with a pseudoaneurysm 1 year after emergency surgery for acute type A aortic dissection. Eight years after the first surgery, she experienced sudden chest pain and presented to the emergency department. Her dyspnea worsened; therefore, echocardiography and three-dimensional computed tomography (3DCT) were performed, and a pseudoaneurysm and shunt to the right ventricle were identified. The medical team attempted to close the shunt with a percutaneous catheter but was unsuccessful, and she was referred to our department for surgical treatment. The pseudoaneurysm originating from the proximal side of the aorta was large (diameter = 55 mm), and echocardiography-gated 3DCT identified the shunt from the pseudoaneurysm to the right ventricle. First, extracorporeal circulation was initiated, and resternotomy was performed. We could not insert the left ventricular venting tube from the right side because of the pseudoaneurysm size. Instead, the tube was inserted from the left atrial appendage. We found a half-circumferential disengaged anastomosis around the proximal anastomosis, which formed the large pseudoaneurysm leading to a fistula in the right ventricle. We closed the fistula and performed a Bentall operation. The patient had a good postoperative course and was discharged on postoperative day 21. She continued treatment for SLE and Sjogren’s syndrome, and her inflammatory reaction improved.

**Conclusions:**

We performed a Bentall operation and fistula closure with resternotomy in a patient with type A aortic dissection with SLE and Sjogren’s syndrome. Multimodal imaging is essential in defining the pseudoaneurysm and the fistula surrounding the anatomy while ensuring their resolution and guiding the approach for operation.

## Background

Pseudoaneurysms after acute aortic dissection are reported to occur in 5–8% of cases [1], and the leading causes of this condition are severe calcification and fragility of the aortic wall, immature surgery, infection, and connective tissue diseases such as Marfan syndrome [2, 3]. In cases of disconnection in the suture line between the artificial graft and native aortic wall, the incidence of larger pseudoaneurysms increases [4]. Furthermore, pseudoaneurysms can increase in size and extend directly into the right ventricle to form a fistula. This complication is infrequent, but cases of fistula formation in the right atrium, right ventricle, and pulmonary artery have been reported [5]. This condition also affects the control of acute heart failure because it causes rapid right heart overload hemodynamics.

Systemic lupus erythematosus (SLE) manifests as an inflammatory response in multiple organs, and the degree of inflammation reflects the severity and course of the disease. Recently, treatment with steroids and immunosuppressive drugs has increased the life expectancy of patients with SLE, revealing significant involvement of the cardiovascular system in the later stages of the disease [6]. Aortic dissection is not a common complication in patients with SLE, but its incidence is reported to be higher in patients with SLE than in age- and sex-matched controls [7]. Moreover, patients with SLE often report difficulties, including sudden back pain, at the time of aortic dissection. However, it is not uncommon for aortic dissection to go undiagnosed until bilateral hemothorax or cardiac tamponade develops [8].

Surgical repair is the primary treatment option for pseudoaneurysms, but the mortality rate is high, and reoperation is often more dangerous than the initial surgery [9]. Furthermore, it is challenging to surgically approach large pseudoaneurysms, and the thoracotomy itself becomes hazardous if it contacts the sternum, superior vena cava, right ventricle, or right atrium. Three-dimensional computed tomography (3DCT) with transesophageal echocardiography and electrocardiography can provide helpful information during preoperative planning.

This study reports a case of a patient with a fistula in the right ventricle and a history of SLE and Sjogren’s syndrome and describes the imaging and surgical procedures performed after acute aortic dissection.

## Case presentation

A 69-year-old woman presented with a chief complaint of exertional dyspnea. She experienced palpitations when climbing the stairs 3 months before her visit to our department, and her dyspnea had worsened thereafter. She experienced thrombocytopenic purpura at the age of 26 years and underwent splenectomy. She was also diagnosed with SLE and Sjogren’s syndrome at the age of 35 years and was treated with prednisolone (10 mg/day) for 1 year for her SLE. After the end of treatment, the SLE was well controlled. She had been on medical therapy for high blood pressure (olmesartan medoxomil, 10 mg/day) and underwent endoscopic treatment for stomach cancer. She had no history of smoking. She was diagnosed with acute aortic dissection (Stanford type A) 10 years before her visit to our department, and hemiarch aortic replacement was performed with an artificial graft. She was noted to have a small pseudoaneurysm at the proximal anastomosis 1 year postoperatively. The surgeon who performed the first surgery did not choose reoperation due to the risk of collagen disease but followed up with the patient every 6 months. The patient had also experienced sudden-onset dyspnea and chest pain 1 year before the current presentation. She was rushed to the same hospital where she underwent her first surgery and was treated for acute heart failure. Contrast-enhanced CT revealed a pseudoaneurysm perforating the right ventricle, and surgical treatment was considered necessary. The reoperation was deemed technically challenging and a high-risk surgery because the pseudoaneurysm was in contact with the sternum. Due to the high risk, fistula closure was attempted with a catheter; however, this was unsuccessful at the first hospital. The treating physician at that hospital recommended surgery at our hospital, leading to the patient's transfer.

Physical examination revealed a height of 160.6 cm and weight of 48.1 kg. Her blood pressure was 110/56 mmHg and heart rate was 66 bpm in sinus rhythm. Her oral medications were furosemide 40 mg/day, tolvaptan 15 mg/day, and bisoprolol fumarate 5 mg/day. Laboratory data were as follows: hemoglobin level, 11.2 g/dL; platelet count, 196 × 109 U/L; total protein level, 7.5 mg/dL; albumin level, 3.9 mg/dL; triglyceride level, 90 mg/dL; low-density lipoprotein cholesterol level, 81 mg/dL; high-density lipoprotein cholesterol level, 75 mg/dL; serum creatinine level, 1.00 mg/dL; hemoglobin A1c (HbA1c) level, 5.7%; brain natriuretic peptide (BNP) level, 718 pg/; C-reactive protein level, 0.15 mg/L; fibrin degradation product (FDP) D-dimer level, 3.2 μg/ml; anti-nuclear antibody positive; anti-Smith antibody negative; double-stranded DNA IgG level, 15 IU/mL; antiphospholipid (antibody) syndrome negative; anti-SS-A/SS-B antibody negative; antiphospholipid antibodies negative; and antineutrophil cytoplasmic antibody (PR3/MPO) titer, 1.0/1.0 U/mL.

**Fig. 1:**
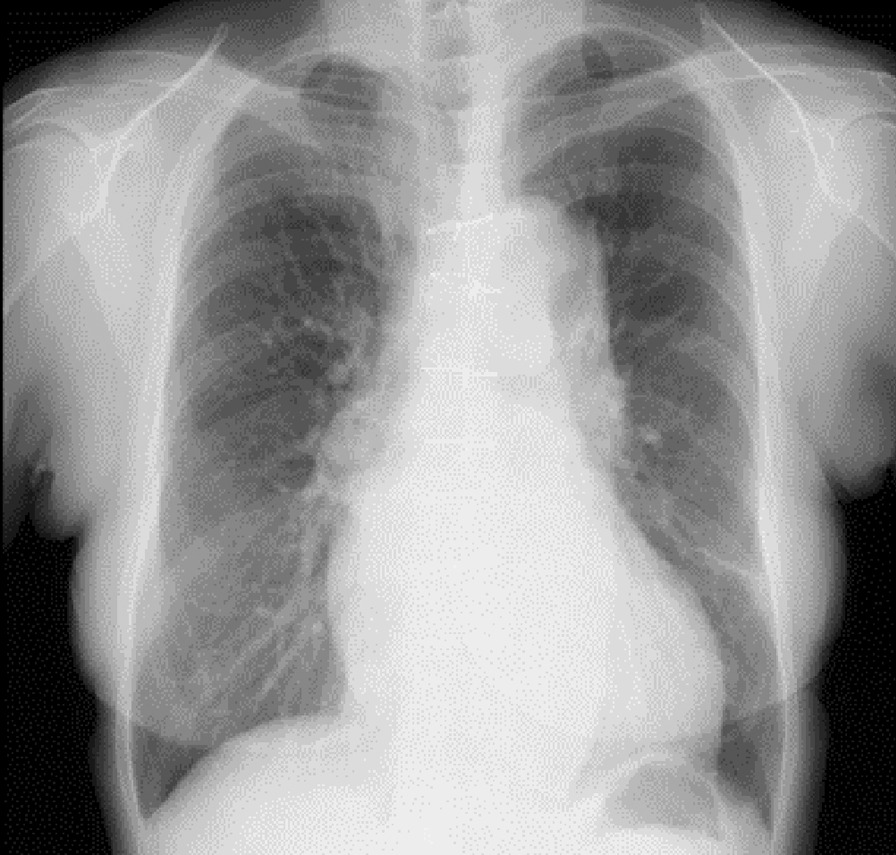
Preoperative chest X-rays. A cardiothoracic ratio of 60% is noted

**Fig. 2:**
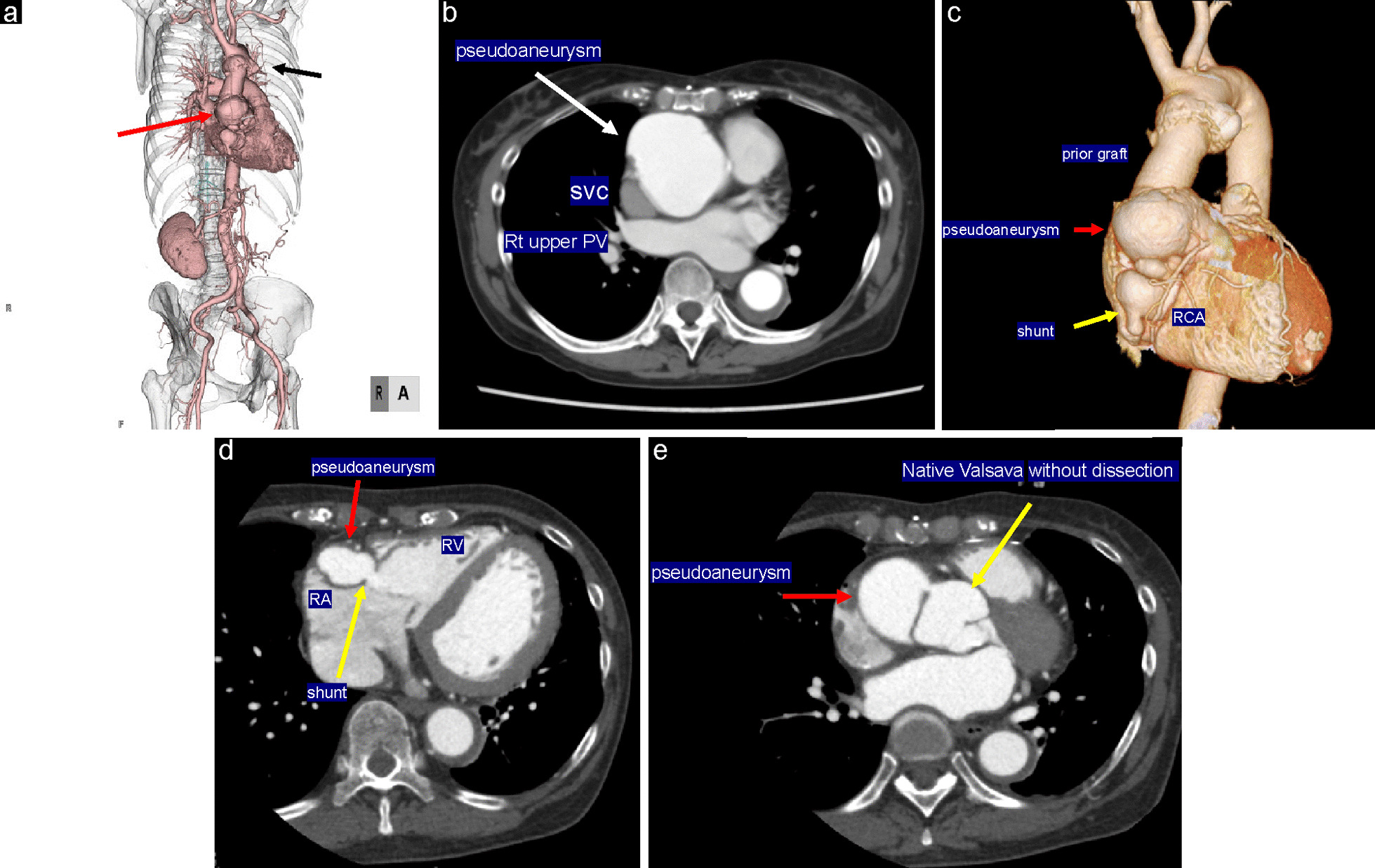
Preoperative 3DCT images. **a** Preoperative 3DCT showing a true saccular aneurysm on the lesser curvature side (black arrow) and a proximal anastomotic pseudoaneurysm (red arrow). **b** The pseudoaneurysm (white arrow) is in close contact with the sternum. **c** ECG-gated 3DCT is used to visualize the pseudoaneurysm (red arrow) and the previously used artificial graft (“prior graft”). The pseudoaneurysm is not in direct contact with the right coronary artery, with a small distance between them. **d** The pseudoaneurysm (red arrow) and shunt (yellow arrow) are near the right heart structures. **e** The Valsalva without the aortic dissection (yellow arrow) is shown with the pseudoaneurysm (red arrow). 3DCT, three-dimensional computed tomography; ECG, echocardiography; SVC, superior vena cava; rt upper PV, right upper pulmonary vein; RA; right atrium. RV; right ventricle; RCA, right coronary artery

**Fig. 3:**
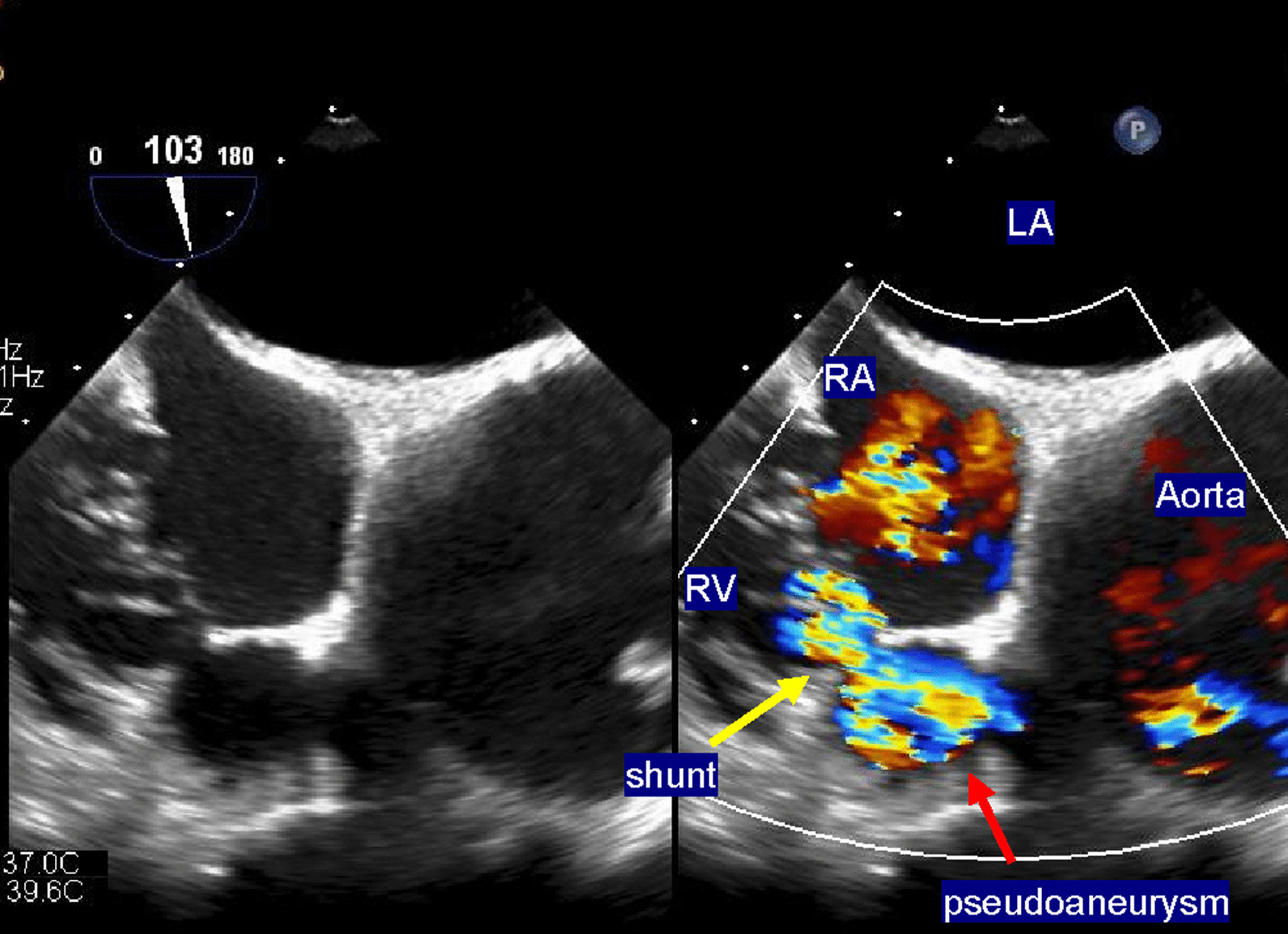
Transesophageal cardiac ultrasonography showing the pseudoaneurysm (red arrow). The shunt (yellow arrow) is indicated. RA, right atrium; RV, right ventricle; LA, left atrium

Electrocardiography revealed normal sinus rhythm. Chest X-ray revealed a cardiothoracic ratio of 60% (Fig. 1), and CT demonstrated a true saccular aneurysm on the lesser curvature side with a proximal arch diameter of 45 mm, a proximal anastomotic pseudoaneurysm of 60 mm, and a Valsalva of 37 mm (Fig. 2). The annulus diameter was 21 mm with mild to moderate aortic valve regurgitation, shunt flow into the right ventricle from this pseudoaneurysm was recognized, and the pulmonary blood flow/systemic blood flow ratio (Qp/Qs) was 1.05 (Fig. 3). Her left ventricular ejection fraction was 68%, with mild tricuspid regurgitation, and the estimated right ventricular pressure was 39 mmHg (risk score: Euro II 20.25%, Japan Score 10.9%). Electrocardiography-gated cardiac 3DCT was performed preoperatively, and the pseudoaneurysm was found to be located in the proximal anastomosis, with the morphology of the Valsalva being preserved. The shunt perforated the right ventricle and was not in contact with the right coronary artery. We found it challenging to dissect the pseudoaneurysm before establishing cardiopulmonary bypass (CPB) in the re-aortic surgery.

**Fig. 4:**
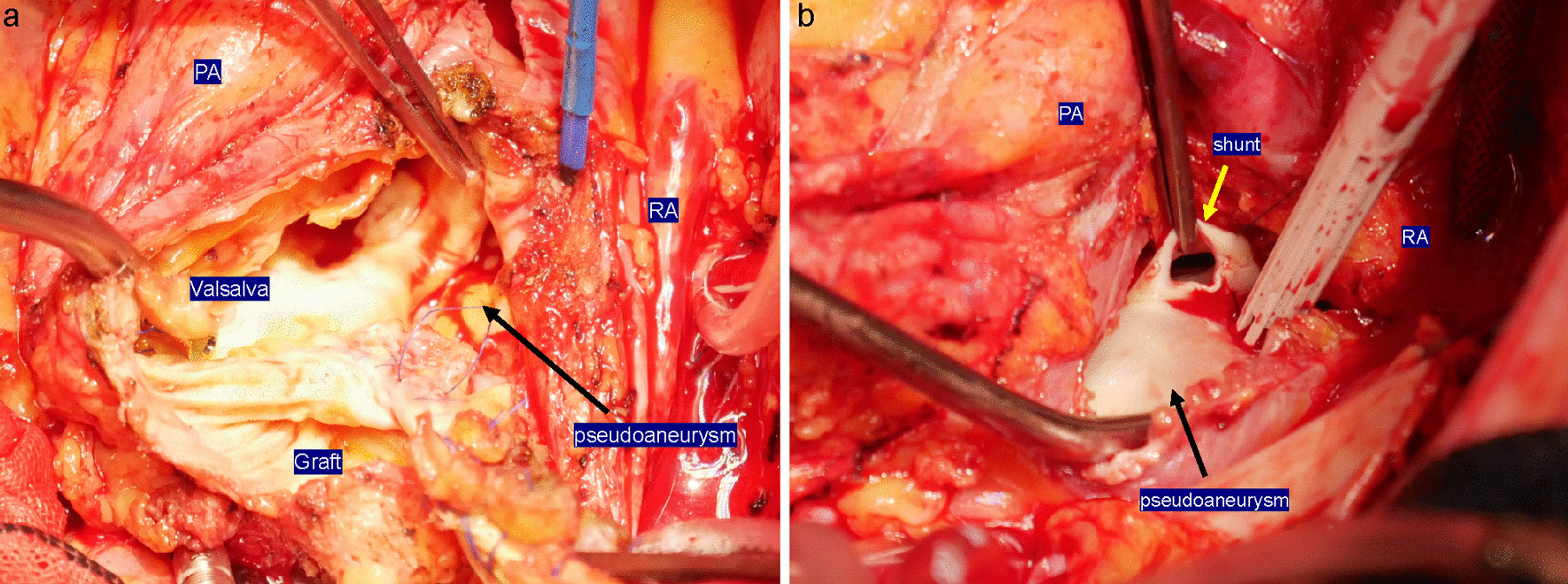
Operative findings. **a** Intraoperative view showing the pseudoaneurysm (black arrow). At the ST junction, the artificial graft and aortic wall are separated by half of the posterior wall. **b** Intraoperative view showing the pseudoaneurysm’s (black arrow) proximity to the shunt (yellow arrow). RA, right atrium; LA, left atrium

**Fig. 5:**
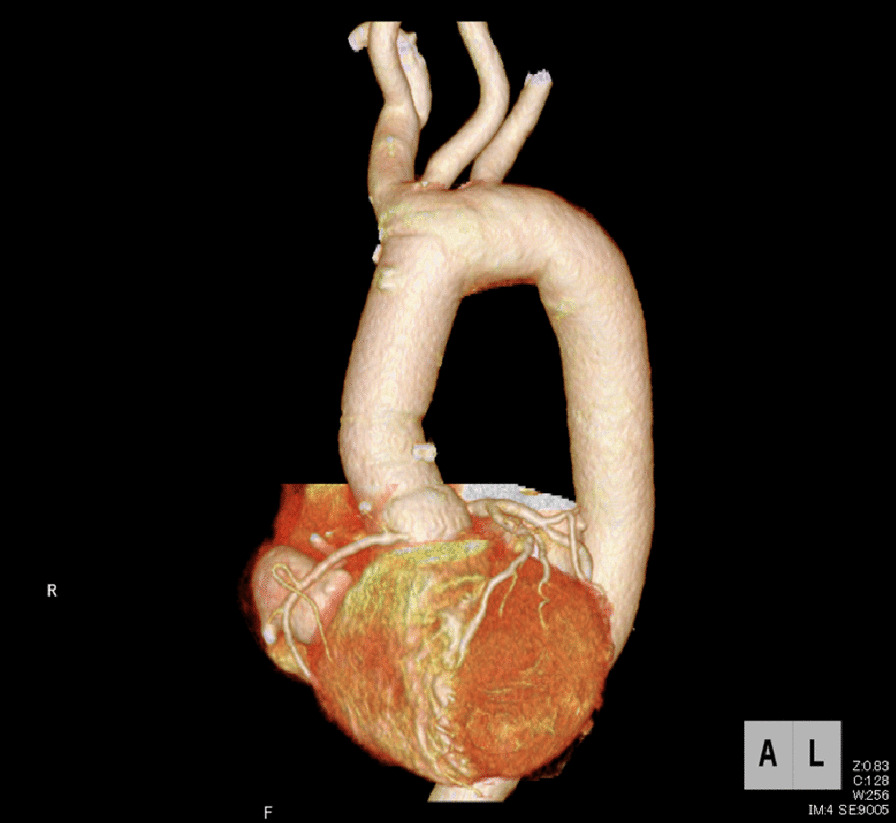
Postoperative ECG-gated 3DCT showing no pseudoaneurysm and a normal right coronary artery. ECG, echocardiography; 3DCT, three-dimensional computed tomography

Sternotomy was performed similarly to the first surgery. Before adhesion detachment, an artificial CPB was established with the inflow on the right subclavian artery and the outflow on the right femoral vein and was then placed into the left atrial venting tube via the left atrial appendage. Initially, we initially planned to vent from the pulmonary artery. However, there were few adhesions on the left side of the heart, and we could quickly identify the left atrial appendage. Moreover, the pulmonary artery had very tight and solid adhesions with the artificial graft, and we thought that the venting tube inserted into the pulmonary artery would interfere with the artificial graft resection. We exfoliated the tissue surrounding the pseudoaneurysm and placed a tape around the distal superior vena cava. We resected the true saccular aneurysm at the distal anastomosis using an open-distal technique. We removed the previous graft and performed distal anastomosis with selective cerebral perfusion under systemic hypothermia. Regarding the strategy for brain protection, the target minimum core temperature was approximately 28 °C, and the circulatory arrest time was 25 min. After distal anastomosis, systemic rewarming was initiated. At the ST junction, the artificial graft and aortic wall were separated by half of the posterior wall (Fig. 4a). We were able to identify a fistula leading from the pseudoaneurysm to the right ventricle (Fig. 4b), which was closed while reinforcing the fistula with a pericardial patch. Because the Valsalva wall was fragile and vulnerable, aortic root reconstruction was performed using the bio-Bentall technique (Inspiris 23 mm: Edwards Lifesciences, Irvine, CA, USA; Gelweave Valsalva 26 mm: Vascutek Ltd., Terumo Aortic, Scotland, UK). The pathological findings of the pseudoaneurysm showed that the aortic intima and media were absent and that only the adventitia was preserved. The operation, CPB, and aortic cross-clamp times were 466, 252, and 189 min, respectively. The respiratory support time was 12 h postoperatively. The patient was discharged from the intensive care unit on postoperative day 2, and rehabilitation was initiated. She experienced postoperative atrial fibrillation; however, no other complications occurred. The hemodynamic performance was excellent without a shunt, and there was no evidence of any pseudoaneurysm on postoperative 3DCT (Fig. 5). One year after the surgery, the patient resumed daily activities effectively, and we found no residual shunt on postoperative echocardiography.

## Discussion and conclusions

In this study, we performed a Bentall operation and fistula closure with resternotomy in a patient with type A aortic dissection with SLE and Sjogren’s syndrome. Pseudoaneurysms at the anastomosis may occur in patients with collagen disease requiring steroid therapy, and if observed, it may lead to shunting of the heart and complications of heart failure. Although reoperation after aortic surgery is challenging, creating an image of the operation in a preliminary manner using multimodal imaging allows the entire surgical team to understand the surgical procedure. Our approach in this case can provide a guideline framework for teams facing such challenging reoperations.

Postoperative pseudoaneurysms of the ascending aorta are associated with periportal extension and high mortality [10]. The actual incidence of pseudoaneurysm after cardiac surgery is unknown but is estimated to be < 0.5% [11]. As the pseudoaneurysm expands, compression of the surrounding tissue structures, infection, rupture or perforation, and a sudden hemodynamic collapse that can turn fatal may occur. The only treatment is surgical repair, but the mortality rate is reportedly as high as 15.4%. Recently, the percutaneous approach has been applied with increasing frequency, particularly in patients at a high surgical risk [12].

Imaging studies can easily identify a pseudoaneurysm in the postoperative period. However, in cases of a sudden onset of heart failure, an immediate diagnosis is required, as in the present case. Transesophageal echocardiography can clearly indicate the origin of the pseudoaneurysm and reveal whether the fistula is penetrating; however, it does not allow for determination of the surgical approach for reoperation. Contrast-enhanced CT provides a better understanding of the pseudoaneurysm location and its relationship with the sternum and surrounding aorta, superior aorta, pulmonary artery, and pulmonary vein. It is an effective tool for determining the surgical approach for reoperation. However, when a pseudoaneurysm forms a shunt in the right ventricle, it is challenging to determine the exact positional relationship between the aortic root and right coronary artery using conventional imaging studies. Echocardiography-gated 3DCT clearly depicts the aortic root and its positional relationship with the coronary origin and right coronary artery, and we believe these factors are essential for preoperative planning. Multimodal imaging is important for revealing the pseudoaneurysm and surrounding anatomy as well as for guiding treatment and ensuring surgical success [13].

It has been reported that four major challenges must be overcome in such a surgery: (1) rupture of the pseudoaneurysm during reopening of the chest, (2) cerebral perfusion during circulatory arrest, (3) ventricular distension due to aortic valve insufficiency, and (4) increased right and left shunt volume during CPB [14].

In cases wherein the sternum is in close contact with the pseudoaneurysm, the sternal incision itself may cause a fatal rupture; therefore, it is imperative to establish CPB before the sternal incision. In the presence of aortic insufficiency or a shunt from a pseudoaneurysm, progressive myocardial protection cannot be injected accurately, and retrograde myocardial protection is adequate. In addition, ventricular fibrillation occurs during the transition to hypothermia, and this condition requires good left ventricular venting because the left ventricle expands rapidly. Further, circulatory arrest by simple hypothermic perfusion should be avoided in aortic regurgitation because of the high likelihood of hypothermia-induced ventricular fibrillation and subsequent ventricular filling. In such situations, the most important concern is how to insert the LV venting tube. However, if there is a large pseudoaneurysm on the right side of the longitudinal wall, the right pulmonary vein cannot be identified, and the right atrium cannot be incised unless the superior vena cava can be identified. In addition, if the adhesion between the artificial graft and the sternum or chest wall is so strong that open chest operation is very difficult, a left ventricular vent may be inserted through the transthoracic wall [15, 16]. In such situations, the venting tube may be placed via the left atrial appendage. In our case, we initially planned to insert the venting tube through the pulmonary artery, but the left side of the heart had almost no adhesions, and the pulmonary artery was highly adherent to the artificial graft; therefore, we inserted the tube through the left atrial appendage. Intraoperative transesophageal ultrasonography is the most effective method for observing this condition. In this situation, the information obtained from intraoperative transesophageal ultrasound can be beneficial.

An increased relative risk of developing aneurysmal lesions has been reported in patients diagnosed with SLE [7, 17] as well as in patients with aortic dissection, with older age, male sex, SLE duration of > 3 years, and hypertension reported as risk factors for both aortic aneurysms and dissection [7]. In terms of pathological findings, histological findings of diffuse aortitis are noted in cases of aortic dissection in patients with SLE, and medial cystic changes and loss of elastic fibers are used to identify the primary lesions [18, 19]. In our study, we believe that the SLE had little effect on the patient because the inflammatory response was negative and stable after 1 year of steroid treatment for the diagnosed SLE. However, the pathological findings included a marked lack of intima and tunica media, indicating that inflammation of the aorta may have existed at the time of surgery.

This study has a limitation in that there was no previous medical history, and the association with SLE or Sjogren’s syndrome is not clear. In our case, the patient abruptly developed a shunt and heart failure, but we could not determine whether the patient would have stabilized if we waited or whether emergency surgery would have been necessary and desirable. In addition, because of failure of the first catheterization, we opted for surgical treatment from the beginning and did not attempt catheterization. We used 3DCT and transesophageal echocardiography to understand the anatomy of the pseudoaneurysm, fistula, and Valsalva wall and to determine the appropriate surgical procedure. Because pseudoaneurysms are particularly susceptible to rupture, the preoperative imaging information was beneficial for removing the surrounding cardiac and aortic tissue.

In conclusion, we performed a Bentall operation and fistula closure with resternotomy in a patient with type A aortic dissection and SLE and Sjogren’s syndrome. A multidisciplinary approach, including a medical doctor’s diagnosis, is essential for accurate surgery.

## Data Availability

The datasets associated with this manuscript are not publicly available but can be made available by the corresponding author upon reasonable request.

## Abbreviations

SLE: Systemic lupus erythematosus
3DCT: Three-dimensional computed tomography
HbA1c: Hemoglobin A1c
BNP: Brain natriuretic peptide
FDP: Fibrin degradation product
CPB: Cardiopulmonary bypass
